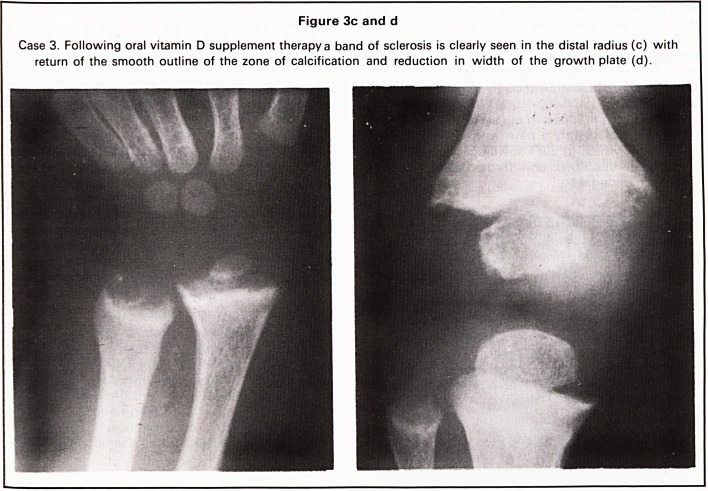# Nutritional Ricketts in Rastafarian Children

**Published:** 1983-04

**Authors:** A. P. Wolinski, R. Nakielny, A. Duncan

**Affiliations:** Department of Diagnostic Radiology, Royal Hospital for Sick Children, Bristol; Department of Diagnostic Radiology, Royal Hospital for Sick Children, Bristol; Department of Diagnostic Radiology, Royal Hospital for Sick Children, Bristol

**Keywords:** Nutritional rickets, Rastafarian infants, 'At-risk' group

## Abstract

Nutritional rickets in children, particularly of Asian immigrants to the U.K., has been well documented over the past 20 years. Its incidence among infants whose parents are Rastafarians of West Indian origin has not previously been described. In a 3-month period four cases of florid nutritional rickets were discovered at this hospital, and a new 'at-risk' group may have been identified.


### Nutritional Rickets in Rastafarian Children

**A. P. Wolinski, R. Nakielny and A. Duncan**
Department of Diagnostic Radiology, Royal Hospital for Sick Children, Bristol

*Keywords*: Nutritional rickets; Rastafarian infants; 'At-risk' group

### ABSTRACT

Nutritional rickets in children, particularly of Asian immigrants to the U.K., has been well documented over the past 20 years. Its incidence among infants whose parents are Rastafarians of West Indian origin has not previously been described. In a 3-month period four cases of florid nutritional rickets were discovered at this hospital, and a new 'at-risk' group may have been identified.

### INTRODUCTION

In recent years the incidence of rickets in the United Kingdom has decreased and various studies have identified 'at-risk' groups in the community (Working Party Report, 1980; Hay, 1979).

Other studies have shown an association between nutritional rickets and a strict vegetarian diet (Dwyer et al., 1979) and have described nutritional rickets in West Indian children (Dawson and Mondle, 1972; Gertner, 1979). This paper describes the incidence of nutritional rickets in four infants whose parents are Rastafarians of West Indian origin.

### PATIENTS

Of seven Rastafarian children seen over a 3-month period, four were found to have florid nutritional rickets. Radiological and biochemical evidence of rickets was present in each case and dietary history confirmed inadequate vitamin D intake.

Following oral vitamin D supplement therapy, the radiological response was assessed.

### CASE REPORTS

### Case 1

An 11-month-old girl presented with a painful right arm following a recent fall. A click was felt at the proximal radioulnar joint. Radiographs of her wrist and chest showed typical rachitic changes (Figure 1a and b). Serum biochemistry revealed a raised alkaline phosphatase (115 kA Units); serum calcium, phosphate were normal. A dietary history was taken and the dietary vitamin D intake was estimated to be 2.2 µg. A course of oral cholecalciferol (3000 Units daily) was commenced. A positive radiological response to therapy was seen after 1 month.

**Figure 1a**

Case 1. Rachitic changes are present at the metaphyses, most marked at the distal ulna.

**Figure 1b**

Case 1. Flaring of the anterior rib ends resulting in the 'rachitic rosary'.

### Case 2

A 13-month-old girl presented with a recent history of dragging her right leg. She had been seen at an earlier age (3 months) with a dislocated hip and was being followed up for this. Clinical examination showed expansion at the ends of the long bones. Radiography of the pelvis showed no dislocation (Figure 2a) but typical rachitic changes were present at the knee (Figure 2b).

**Figure 2a**

Case 2. Pelvis shows no evidence of dislocation of either hip.

**Figure 2b**

Case 2. Knee showing marked rachitic changes in the distal femur and proximal tibia and fibula.

Serum biochemistry revealed a raised alkaline phosphate (148 kA Units) but was otherwise normal.

A course of oral cholecalciferol (3000 Units daily) was commenced and a positive radiological response was seen after 1 month.

### Case 3

A 13-month-old girl presented with a 24-hour history of inability to move her left arm, but there was no

**Figure 3a and b**

Case 3. Marked rachitic changes are present in the wrist and knee before treatment.

history of recent trauma. No limitation of arm movement was present on examination but there was bony expansion at the wrists and knees and a minor degree of tibial bowing. Typical metaphyseal changes of rickets were present in the wrist and knee (Figure 3a and b). Serum biochemistry showed the alkaline phosphatase to be greater than 40 kA Units but was otherwise normal.

A dietary history was taken and the daily dietary vitamin D intake was estimated to be 2.78 µg.

A course of oral cholecalciferol (3000 Units daily) was commenced and radiographs taken 1 month later (Figure 3c and d) showed a positive response to therapy.

**Case 4**

A 22-month-old girl was seen since she was the step-sister of Case 1. She was asymptomatic and the only findings on examination were slight expansion at the ends of her long bones. Rachitic changes were present on radiographs of her wrist and knee. Serum biochemistry revealed her alkaline phosphate to be elevated (145 kA Units) but was otherwise normal.

She was commenced on oral cholecalciferol (3000 Units daily) and a positive radiological response to therapy was present after one month.

### DISCUSSION

During the 1960s and 1970s the incidence of rickets in the United Kingdom showed a rise with a subsequent fall (Benson, 1963; Ford et al., 1976; Goet et al., 1976, 1979). Children of Asian immigrants were identified as the main 'at-risk' group (Working Party Report, 1980) although some cases were seen among West Indian children and in children from other ethnic groups (Dawson and Mondle, 1972; Gertner, 1979).

Radiology has an important role in the diagnosis of rickets and may be used to assess response to therapy. The radiological changes on which a diagnosis of rickets can be made were defined by the Working Party Report (1980) as:

1. Flaring and cupping of the metaphyses at the growing ends of long bones.

**Figure 3c and d**

Case 3. Following oral vitamin D supplement therapy a band of sclerosis is clearly seen in the distal radius (c) with return of the smooth outline of the zone of calcification and reduction in width of the growth plate (d).

2. Widening of the cartilaginous growth plate in a longitudinal direction.

3. Loss of the smooth, even zone of calcification and its replacement by a ragged one.

In all four of our cases, all these changes were present at the wrist and knee and were also seen at the anterior ends of ribs in Case 1, the so-called 'rachitic rosary' (Figure 1b).

Radiological signs indicating a positive response to therapy in our study were:

1. The appearance of a metaphyseal band of sclerosis.

2. Reduction in width of the cartilaginous growth plate.

3. Return of the smooth even zone of calcification.

Studies have shown that low dietary vitamin D is not peculiar to Asians but is also to be found in whites and West Indians (Working Party Report 1980) and have also shown the association of vegetarian diets, with rickets, particularly where little or no meat, milk, fats or oils are taken (Edidin et al., 1980; Bacharach, 1979). Details of dietary intakes were obtained in two of our infants. The children were breast fed into the second half of the first year of life and were then started on an 'I-tal' type vegan diet which comprises little or no milk, meat or fats but is mainly vegetable. In both cases the daily intakes of 2.2 μg and 2.78 μg vitamin D fell well short of the recommended 10 μg daily minimum. Calcium intake was adequate in both children.

The association of nutritional rickets, prolonged breast feeding and vegan diet was described by Edidin (Edidin et al., 1980) who also found a variable mode of presentation ranging from upper respiratory tract infection to non-weight-bearing, limping and pain after a fall. A similar variety of presentation was present in our patients.

One of our patients was the asymptomatic step-sister of the first patient and similar familial occurrences have been described (Haider, 1974).

Rickets has been documented in Jamaica (Miller and Chuktan, 1976) as well as in West Indians in the United Kingdom. In the Jamaican study, nine cases were found over a 5-year period and all were children over 3 years of age.

Our study showed an earlier presenting age, all four cases were under 2 years old.

The Jamaican study made no mention of vegetarian diet or of Rastafarian culture, and indeed fish appears to be an important part of the diet in Jamaica.

The Rastafarian group of West Indians are a sect who believe that Haile Selassie was their leader and that they will eventually return to their land of Ethiopia. The habits and customs of this sect vary slightly but they are all vegetarians. The eating of meat, fish and fats is forbidden. Most adhere to the strict dietary limitations although some occasionally eat cheese.

Although the numbers in our study are small, they could serve to identify a new 'at-risk' group in the community, which to our knowledge has not previously been described and further, larger studies should be undertaken.

### ACKNOWLEDGEMENTS

The authors are grateful to Drs. P. Ward and J. James for their cooperation in the study; Mr. J. Hancock for photography and to Miss J. Hugh for typing the manuscript.

### REFERENCES

BACHARACH, S. et al. (1979). An outbreak of vitamin D deficient rickets in a susceptible population. *Paediatrics* **64**, 871–877.

BENSON, P. F. et al. (1963) Rickets in immigrant children in London. *B.M.J.* **1**, 1054–1056.

DAWSON, K. P. and MONDHE, M. S. (1972) Nutritional rickets among the immigrant population of Bradford. *Practitioner* **208**, 789.

DWYER, J. T. et al. (1979) Risk of nutritional rickets among vegetarian children. *Am.J.Dis.Child.* **133**, 134–140.

EDIDIN, D. V. et al. (1980) Breast feeding, nutritional rickets, vitamin D deficiency and fad diets. *Paediatrics* **65**, 232–235.

FORD, J. A. et al. (1976) Clinical and subclinical vitamin D deficiency in Bradford children. *Arch.Dis.Child.* **51**, 929–943.

GERTNER, J. M. (1979) Preventing nutritional rickets. *Lancet* **I**, 257.

GOEL, K. M. et al. (1976) Florid and subclinical rickets among immigrant children in Glasgow. *Lancet* **I**, 1141–1145.

GOEL, K. M. et al. (1979) Reduced prevalence of rickets in Asian children in Glasgow. *Lancet* **II**, 405–407.

HAIDER, S. A. (1974) Screening for rickets. *B.M.J.* **3**, 688–689.

HAY, A. (1979) Rickets under control again in the U.K. *Nature* **279**, 749.

MILLER, C. G. and CHUKTAN W. (1976) Vitamin D deficiency rickets in Jamaican children. *Arch.Dis.Child.* **51** (3), 214–218.

WORKING PARTY REPORT on FORTIFICATION of FOOD with VITAMIN D (1980) Rickets and osteomalacia. *Rep.Health Soc.Subj.(London)* **19**, 1–66.

## Figures and Tables

**Figure 1a f1:**
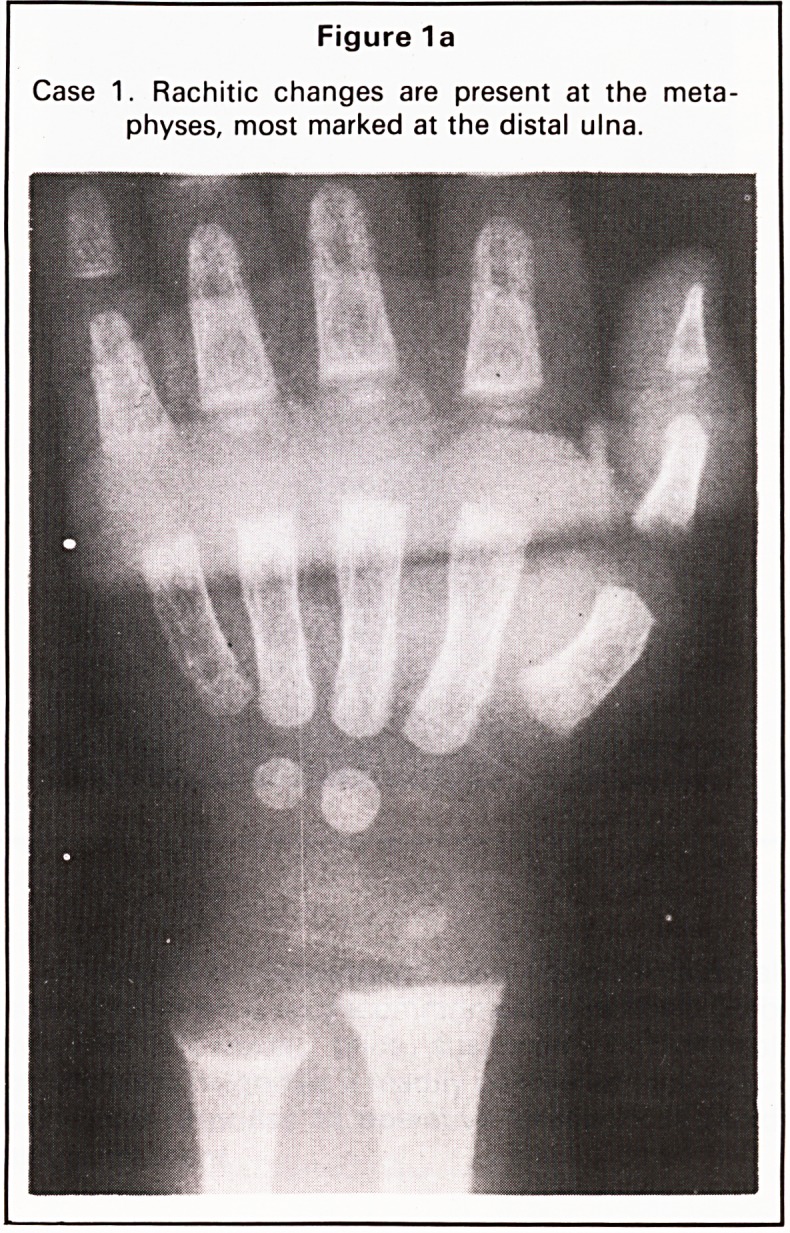


**Figure 1b f2:**
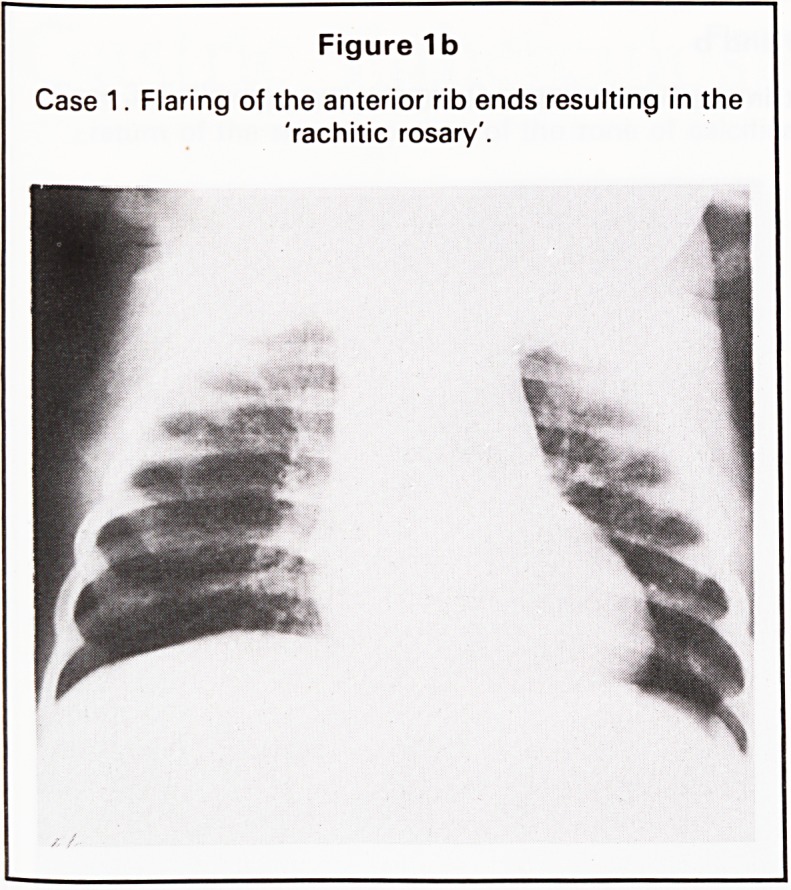


**Figure 2a f3:**
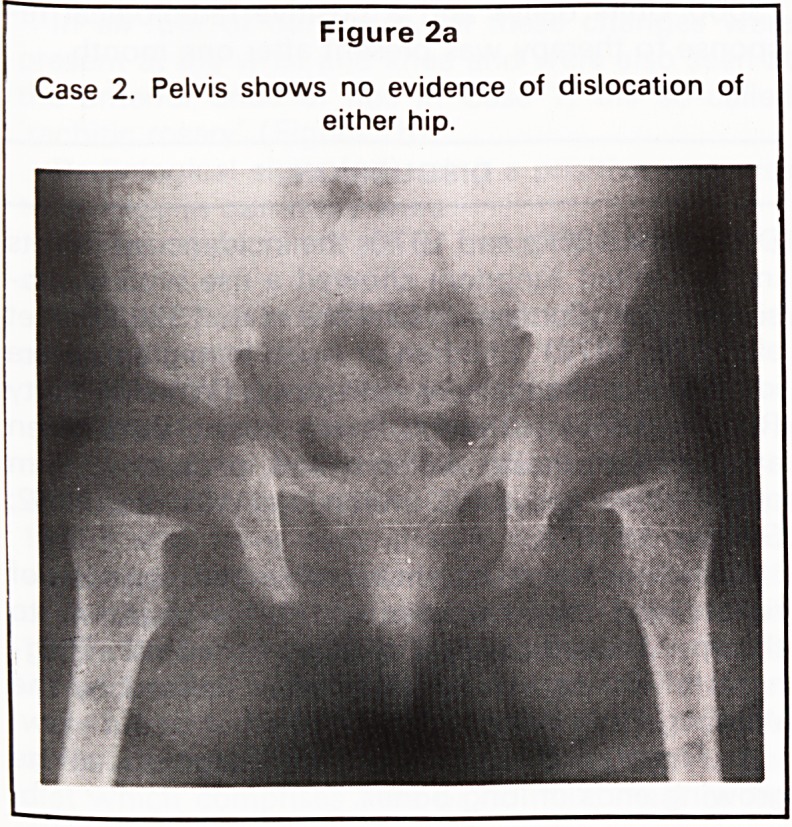


**Figure 2b f4:**
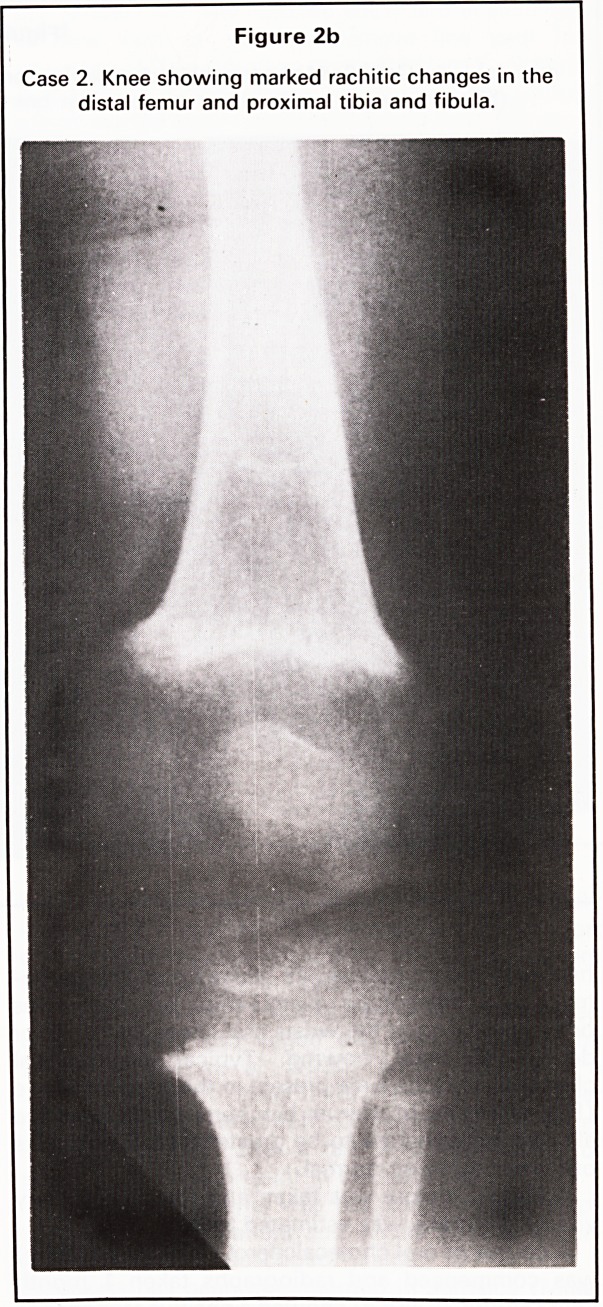


**Figure 3a and b f5:**
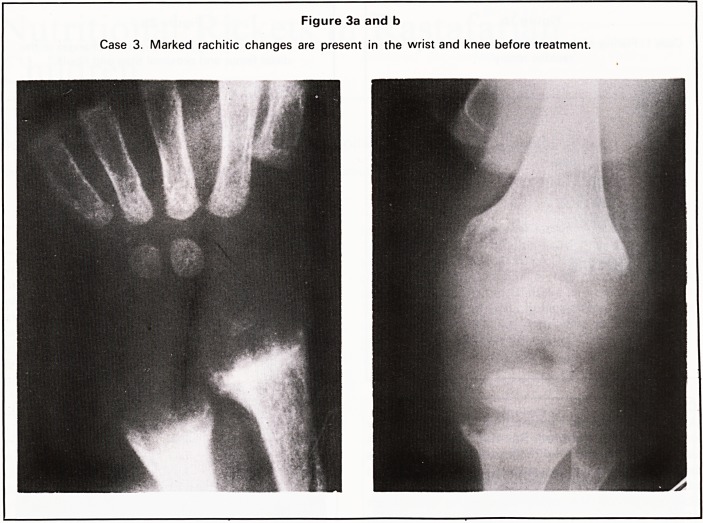


**Figure 3c and d f6:**